# Colonic Dieulafoy's Lesion: A Rare Cause of Lower Gastrointestinal Hemorrhage and Review of Endoscopic Management

**DOI:** 10.1155/2014/436293

**Published:** 2014-10-19

**Authors:** Christopher Ma, Rajveer Hundal, Edwin J. Cheng

**Affiliations:** ^1^Department of Internal Medicine, University of Alberta, 13-103 Clinical Sciences Building, Edmonton, AB, Canada T6G 2G3; ^2^Division of Gastroenterology, University of Calgary, Teaching Research Wellness Building, 3280 Hospital Drive NW, Calgary, AB, Canada T2N 4Z6

## Abstract

Dieulafoy's lesions are a rare cause of gastrointestinal hemorrhage. Extragastric Dieulafoy's lesions are even more uncommon. We report the case of a 75-year-old woman who presented with gastrointestinal bleeding from a transverse colonic Dieulafoy's lesion. She presented with two episodes of melena followed by one episode of fresh blood per rectum. In addition, there was associated presyncope and anemia (hemoglobin 69 g/L) in the setting of supratherapeutic warfarin anticoagulation (INR 6.2) for nonvalvular atrial fibrillation. Esophagogastroduodenoscopy was negative for an upper GI source of bleeding but on colonoscopy an actively oozing Dieulafoy's lesion was identified in the transverse colon. Bipolar cautery and hemostatic endoclips were applied to achieve hemostasis. Clinicians should consider this rare entity as a potential cause of potentially life-threatening lower gastrointestinal bleeding and we review the endoscopic modalities effective for managing colonic Dieulafoy's lesions.

## 1. Introduction

Dieulafoy's lesions (also known as caliber persistent artery, submucosal arterial malformation, or solitary exulceration simplex) are an uncommon cause of gastrointestinal hemorrhage. They are defined by a dilated aberrant submucosal vessel that does not undergo normal distal branching or tapering and subsequently protrudes through a minute defect in the overlying mucosa but without primary mucosal ulceration [1]. Histologically, there is no obvious abnormality in the arterial wall and there is no evidence of vasculitis or arteriovenous shunting [2]. Dieulafoy's lesions are relatively rare: reported incidence as a cause for acute gastrointestinal bleeding is <2% [3, 4]. Gastric Dieulafoy's lesions are the most common, accounting for over 70% of cases, and are typically found in the proximal stomach along the lesser curvature near the esophagogastric junction. In contrast, colonic Dieulafoy's lesions are extremely rare.

Here, we present the case of a 75-year-old woman who developed a lower gastrointestinal bleed secondary to a transverse colonic Dieulafoy's lesion. This case highlights the importance of both careful endoscopic evaluation in investigating gastrointestinal hemorrhage and consideration of rare entities in the differential diagnosis of common clinical presentations. Finally, we review the literature of diagnostic and therapeutic measures previously utilized for identifying and managing colonic Dieulafoy's lesions.

## 2. Case Report

A 75-year-old female presented to hospital with two episodes of melena followed by one episode of moderate volume bright red blood per rectum. She had multiple medical comorbidities including coronary artery disease with previous ST-elevation myocardial infarction complicated by congestive heart failure, diabetes, hypertension, and gastroesophageal reflux disease. She was also known to be in persistent atrial fibrillation, complicated by tachy-brady syndrome requiring pacemaker insertion in 2012 and ongoing anticoagulation with warfarin.

The patient had no previous history of gastrointestinal hemorrhage prior to presentation. Associated with the bleeding, she endorsed presyncopal symptoms but was hemodynamically stable and denied hematemesis. There was no associated nausea, vomiting, diarrhea, or abdominal pain. Initial examination found a benign, nontender, nonperitonitic, nondistended abdomen and rectal examination demonstrated residual melena stool. Initial investigations were significant for severe anemia, with hemoglobin of 69 g/L (baseline hemoglobin > 100 g/L from one year prior), and supratherapeutic warfarin anticoagulation with international normalized ratio (INR) of 6.2. In the emergency department, the patient was supportively managed with intravenous crystalloid fluid and two units of packed red blood cell transfusion. Her anticoagulation was reversed with intravenous vitamin K and transfusion of one unit of fresh frozen plasma.

**Figure 1:**
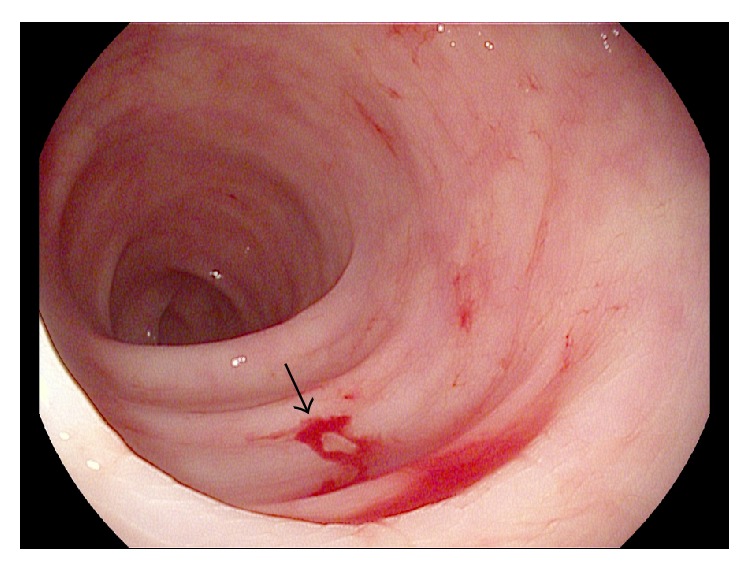
Presence of active oozing blood from transverse colonic Dieulafoy's lesion. Arrow indicates source of bleeding.

**Figure 2:**
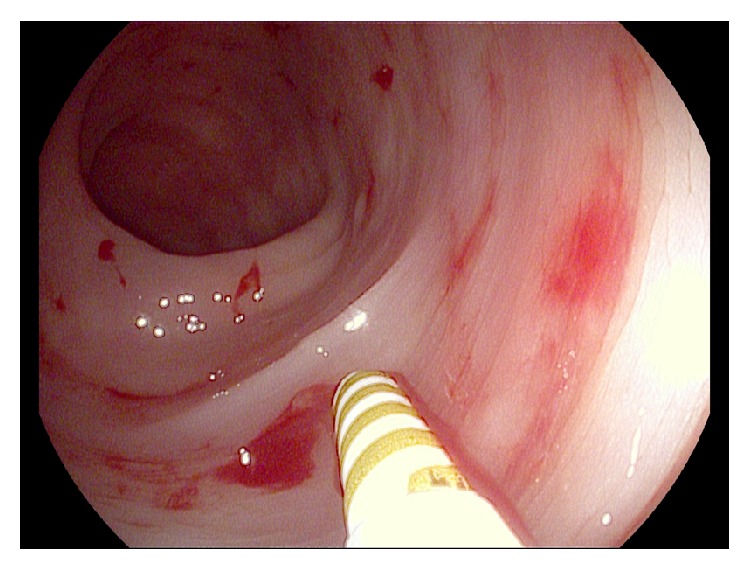
Application of bipolar cautery using 7-French probe to colonic Dieulafoy's lesion.

**Figure 3:**
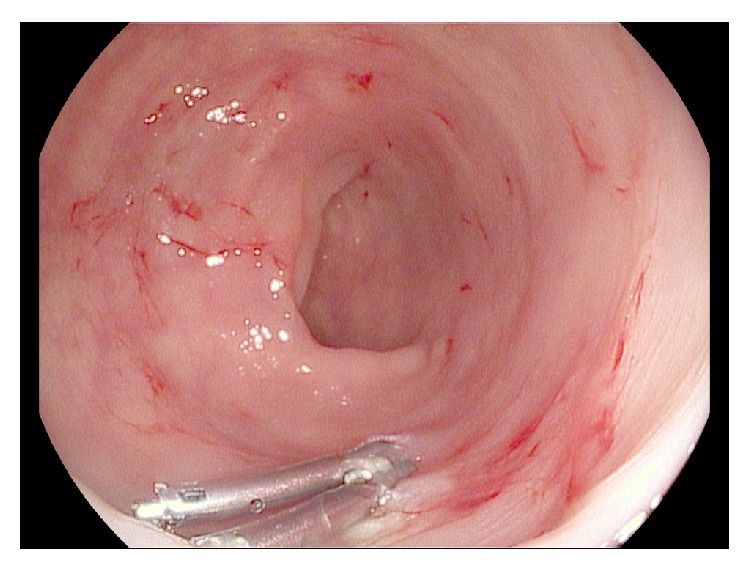
Application of hemostatic endoclip to colonic Dieulafoy's lesion after bipolar cautery, achieving hemostasis.

Esophagogastroduodenoscopy was normal with no evidence of active bleeding. However, on ileocolonoscopy, a large overlying fresh blood clot was visualized in the transverse colon. After disruption of the clot with irrigation, an actively oozing Dieulafoy's lesion was visualized (Figure 1). Bipolar cautery with a 7-French probe was applied (Figure 2) and, subsequently, two hemostatic endoclips were deployed at the bleeding site (Figure 3), achieving hemostasis. The patient did not experience any immediate or delayed postendoscopy complications and she had no clinical or laboratory evidence of ongoing or recurrent bleeding.

## 3. Discussion

First reported in 1985 by Barbier et al. [5], colonic Dieulafoy's lesions are an uncommon cause of lower gastrointestinal bleeding but present unique diagnostic and therapeutic challenges to clinicians. The identification and management of colonic Dieulafoy's lesions have evolved over the last 30 years, particularly with advances in endoscopy. Here, we report the case of a transverse colonic Dieulafoy's lesion and review previously utilized endoscopic measures for management of this rare condition.

Dieulafoy's lesions typically present with painless large volume bleeding, making them clinically difficult to distinguish from other causes of lower gastrointestinal bleeding such as arteriovenous malformations or diverticular hemorrhage. As with our case, bleeding risk is increased in patients with medical comorbidities such as hypertension or cardiovascular disease and in patients on concurrent anticoagulation [6]. Extragastric Dieulafoy's lesions are extremely rare but have been reported throughout the entire colon, including cecum [7, 8], ascending colon [9, 10], descending and sigmoid colon [11], and the rectum [12–15]. In a review by Baxter and Aly of 45 case reports, only 2% of Dieulafoy's lesions were identified in the colon [4].

Although capable of presenting with massive hemorrhage, Dieulafoy's lesions can be challenging to diagnose. In the era before readily available colonoscopy, direct mesenteric angiography was often required for localization of bleeding [7, 9]. Most lesions can now be identified by direct endoscopic visualization, but they may be subtle and easily overlooked. Sensitivity of endoscopy may be further limited in the setting of poor bowel preparation or obscured visual field due to high volume bleeding. As with other presentations of obscure lower gastrointestinal bleeding, CT angiography [15] and red blood cell scintigraphy have also been previously utilized for diagnosis [16].

Management of colonic Dieulafoy's lesions has evolved to become predominantly endoscopic. Early reported cases were treated surgically with partial colectomy, primarily in the setting of uncontrollable, life-threatening, lower gastrointestinal hemorrhage [5, 7, 9, 11]. However, there is now a growing body of evidence for endoscopically achieved hemostasis: 90% of lesions can be controlled with endoscopy [4]. The most commonly used endoscopic measures include epinephrine injection [8, 12] and endoscopic clipping [17–19], and both modalities have previously been shown to achieve immediate and sustained hemostasis to at least six months after hemorrhage. Other therapeutic modalities, including thermocoagulation [20], argon plasma coagulation [15], and endoscopic sclerotherapy [21], have also been utilized effectively, although they are less frequently employed. Several authors have also described the combination of multiple endoscopic techniques to achieve hemostasis, including clipping, adrenaline injection, and laser coagulation [22].

As with gastric Dieulafoy's lesions, rebleeding has been described from colonic sites. Although repeated endoscopic measures have been utilized, rescue therapy with angiographic embolization [14] or surgery [13] may be required. Selective angiography is indicated in patients failing endoscopic therapy, for lesions beyond the reach of therapeutic endoscopy, or in poor candidates for surgery [4]. Extrapolating from literature of gastric Dieulafoy's actively spurting arterial bleeding and use of nonsteroidal anti-inflammatory drugs or anticoagulants may portend higher risk of recurrent hemorrhage [23]. In challenging cases, such as in our patient who was supratherapeutically anticoagulated on warfarin, there is some limited evidence to suggest that combination endoscopic therapy may be more effective; in a review of 29 cases of bleeding Dieulafoy's lesions, Jamanca-Poma et al. found that 69% of cases were managed with a combination of endoscopic techniques, and, although limited by small sample size, combination endoscopic therapy prevented bleeding recurrence compared to adrenaline monotherapy (OR 0.14, 95% CI: 0.19–0.99) [24]. Combined approach with embolization and endoscopy can also be attempted; one report in the literature has even described “adjuvant” arterial embolization used to reduce blood flow in a hemorrhaging duodenal Dieulafoy's lesion with subsequent combination endoscopic laser coagulation as a last resort option [25].

In conclusion, although rare, colonic Dieulafoy's lesions can be a cause of life-threatening hemorrhage that should be considered in the differential diagnosis of lower gastrointestinal bleeding. They are important to recognize as they can be effectively managed using a number of endoscopic techniques, including epinephrine injection, clipping, or thermocoagulation.
